# Sensommatic: an efficient pipeline to mine and predict sensory receptor genes in the era of reference-quality genomes

**DOI:** 10.1093/bioinformatics/btae040

**Published:** 2024-01-23

**Authors:** Louise Ryan, Colleen Lawless, Graham M Hughes

**Affiliations:** School of Biology and Environmental Science, University College Dublin, Belfield, Dublin 4, Ireland; School of Biology and Environmental Science, University College Dublin, Belfield, Dublin 4, Ireland; School of Biology and Environmental Science, University College Dublin, Belfield, Dublin 4, Ireland

## Abstract

**Summary:**

Sensory receptor gene families have undergone extensive expansion and loss across vertebrate evolution, leading to significant variation in receptor counts between species. However, due to their species-specific nature, conventional reference-based annotation tools often underestimate the true number of sensory receptors in a given species. While there has been an exponential increase in the taxonomic diversity of publicly available genome assemblies in recent years, only ∼30% of vertebrate species on the NCBI database are currently annotated. To overcome these limitations, we developed ‘Sensommatic’, an automated and accessible sensory receptor annotation pipeline. Sensommatic implements BLAST and AUGUSTUS to mine and predict sensory receptor genes from whole genome assemblies, adopting a one-to-many gene mapping approach. While designed for vertebrates, Sensommatic can be extended to run on non-vertebrate species by generating customized reference files, making it a scalable and generalizable tool.

**Availability and implementation:**

Source code and associated files are available at: https://github.com/GMHughes/Sensommatic

## 1 Introduction

Sensory perception enables organisms to perceive and interact with their environment. Multiple sensory modes exist, including chemoreception (taste, olfaction, and pheromone detection) and vision. At the molecular level, chemosensory perception and vision are mediated by G-protein coupled receptors (GPCRs), which undergo a conformational change in response to sensory stimuli (Julius and Nathans 2011). Examples of GPCR sensory subfamilies include opsins (OPNs/RHO; vision), vomeronasal receptors (VN1Rs/VN2Rs; pheromone detection), taste receptors (TAS1Rs/TAS2Rs), trace-amine-associated receptors (TAARs), and olfactory receptors (ORs; olfaction).

Chemoreceptor gene families have undergone extensive expansion and loss throughout vertebrate evolution (Shi and Zhang 2009), often as a consequence of unique environmental adaptations (Hayden et al. 2010). This is particularly true in mammals, where receptor counts vary drastically between species (∼2500 functional ORs in the African elephant compared to 60 in the common bottle nose dolphin; Hughes *et al.* 2018). As these receptor counts are often species-specific, conventional reference-based annotation tools, which attempt to predict one-to-one orthologues using a closely related reference species as a guide, will often underestimate the total number of sensory receptors in a given species. Hence, a ‘one-to-many’ mapping approach is required for sensory receptor gene mining.

The number of available genome assemblies has increased exponentially in recent years due to the rapid advancement of sequencing technologies. Long read sequencing in particular has greatly facilitated genome assembly, resulting in reference quality and telomere-to-telomere (T2T) genomes (Nurk *et al.* 2022). Moreover, consortia such as the Vertebrate Genomes Project (Rhie *et al.* 2021), Bat1K (Teeling *et al.* 2018, Jebb *et al.* 2020), ERGA (Formenti *et al.* 2022), and Zoonomia (Genereux *et al.* 2020), promise to further increase the taxonomic diversity of assemblies in the near future. There are currently over 2700 genome assemblies available for vertebrate species, however, annotations are only available for ∼30% of these. Given that sensory receptor genes are underestimated in these annotations, there is a clear need for a sensory receptor gene mining tool to fully exploit this wealth of genomics data. We therefore present ‘Sensommatic’, a fully automated, fast, and accessible sensory receptor gene mining and annotation tool, suitable for the era of reference-quality genomes.

## 2 Methods

**Figure 1.**
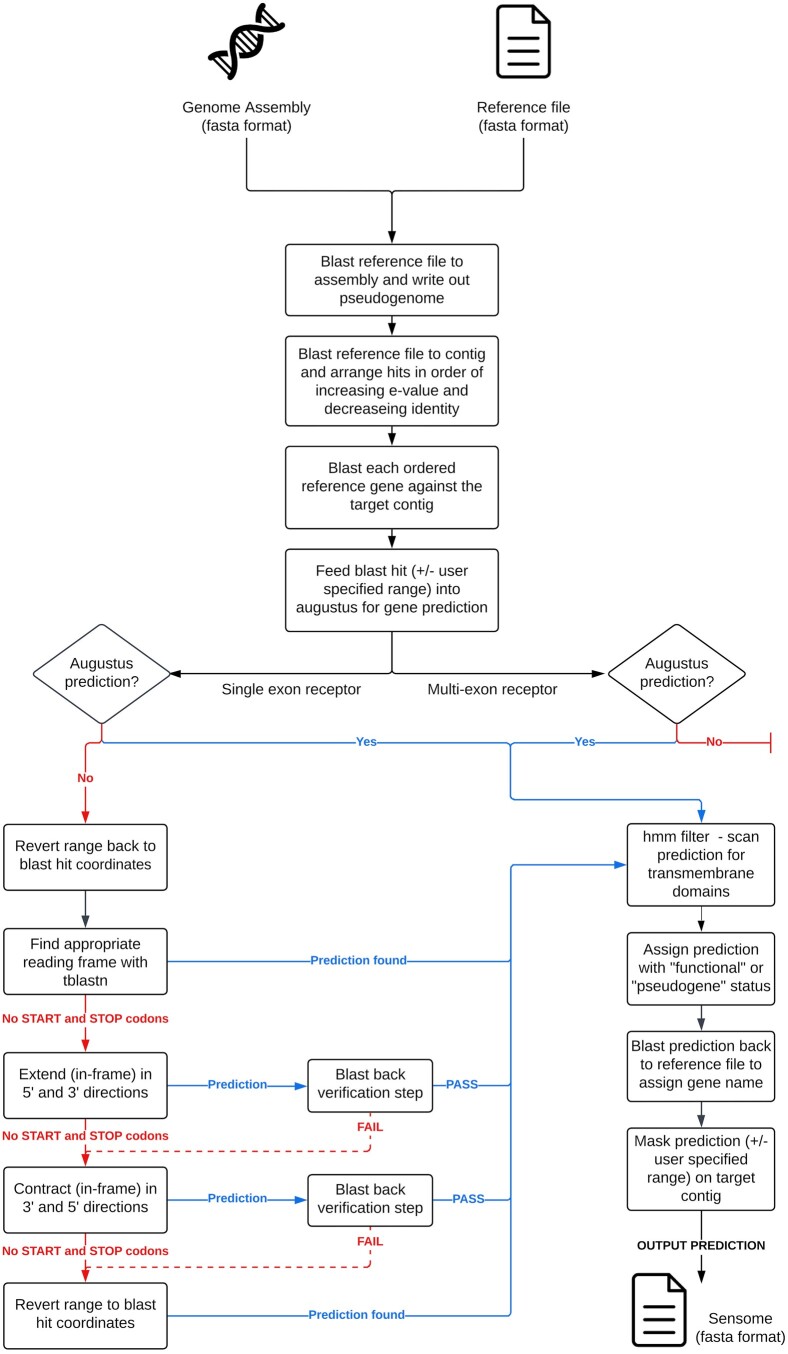
Flowchart illustrating the serial steps implemented in the Sensommatic pipeline.

Sensommatic implements BLAST (Altschul *et al.* 1990) and AUGUSTUS (Stanke *et al.* 2004) to mine and predict sensory receptor genes from whole genome assemblies (Fig. 1). It requires two input files: the genome assembly of a target species and a reference file containing homologous receptors from a set of closely related species. Specifically, genes encoding ORs, TAS1Rs/TAS2Rs, VN1Rs/VN2Rs, TAARs, and OPNs/RHO are represented in the reference file. We provide reference files for each class of vertebrates, including mammals, amphibians, reptiles, birds, and fish. The pipeline outputs a single fasta file containing gene predictions for each sensory receptor family.

### 2.1 Generation of a ‘pseudogenome’ to improve run times

To improve run times for lower-quality genomes, Sensommatic initially performs a BLASTN search of all reference receptors against the genome assembly, using the default BLASTN search parameters. Contigs with significant hits are written to a ‘pseudogenome’ file. To further optimize speed, users can opt to implement the ‘esl-sfetch’ tool from the HMMER easel mini-apps suite (Eddy 2011). If specified, Sensommatic utilizes the ‘esl-sfetch’ genome indexing tool to efficiently access and output contigs.

### 2.2 Mining sensory receptor genes with BLASTN

Using the reference file, BLASTN searches are performed per contig. Hits are arranged by increasing e-value and decreasing identity. For each ordered hit, the identified region is fed into AUGUSTUS for gene prediction. The extended range of nucleotides added to the 3’ and 5’ ends of each hit is specified by the user (default range of 2500 bp for single-exon genes). To account for the larger region occupied, Sensommatic uses a default range of 50 kb for multi-exon genes. Complementary sequences are determined for hits on the reverse-strand.

### 2.3 Gene prediction with AUGUSTUS

Sensommatic applies ‘blat2hints’ in AUGUSTUS to generate hints from the reference file, aiding gene prediction. Only the top five BLASTN hits from the reference file are used to generate hints for OPNs and TAS1Rs as including more reduced prediction accuracy for these specific gene families (owing to their multi-exon composition). The ‘intronless’ AUGUSTUS gene model is applied when predicting single exon sensory receptors.

### 2.4 Predicting single-exon genes

If AUGUSTUS fails to predict a single-exon gene, Sensommatic searches for the appropriate open reading frame (ORF) using TBLASTN. The prediction is checked for start and stop codons at the 5’ and 3’ ends, respectively. If undetected, Sensommatic extends the sequence in-frame to search for the start and end positions (Supplementary Fig. S1). If an ORF is identified, a ‘blastback’ verification step is carried out to compare the prediction to the reference gene. Predictions sharing over 60% coverage and 50% identity with the reference post-extension, are considered valid. If these thresholds are not met, or the start and end positions are not identified within the extension limit, an internal search for the start and stop codons is performed followed by an additional ‘blastback’ verification step (Supplementary Fig. S2). If the prediction fails this final checkpoint, the sequence defaults to the original BLASTN coordinates and is included for downstream analysis.

### 2.5 Validation of predictions with HMMER

Sensommatic implements HMMER to scan predictions for the presence of the characteristic seven transmembrane domains using profile hidden Markov models (HMMs) for Class A and Class C GPCR sensory receptors (Eddy 2011). Predictions lacking transmembrane domains are filtered out to prevent the inclusion of false-positive hits that may have arisen during initial BLASTN searches.

### 2.6 Classification of sensory receptor genes

Predictions are classified as ‘functional’, or ‘pseudogene’ based on the presence of in-frame stop codons and prediction length (860 bp for functionality by default). This modifiable threshold was chosen based on the minimum length required for the seven-transmembrane domain. A shorter length may be more appropriate for low-quality genome assemblies where genes are fragmented by contig boundaries. Predictions are assigned gene names, and hence are classified into sensory receptor families, based on the corresponding top hit in the reference file. Genomic locus coordinates and contig labels are included in the fasta header of each prediction.

### 2.7 Masking predicted regions

To prevent BLASTN hits repeatedly mapping to previously annotated regions of the contig, nucleotides in the predicted range are masked with an equivalent number of ‘N’ sites. By default, a range of 750 bp upstream and downstream of each prediction is also masked to minimize the occurrence of single loci being annotated as multiple genes.

### 2.8 Benchmarking pipeline performance

To assess Sensommatic’s performance, we conducted tests on fourteen vertebrate species: *Homo sapiens* (human), *Pan troglodytes* (chimpanzee), *Pongo abelii* (orangutan), *Mus musculus* (mouse), *Rattus norvegicus* (brown rat), *Canis lupus familiaris* (dog), *Bos taurus* (cow), *Equus caballus* (horse), *Monodelphis domestica* (opossum), *Ornithorhynchus anatinus* (platypus), *Xenopus laevis* (African clawed frog), *Anolis carolinensis* (green anole), *Anas platyrhynchos* (mallard duck), and *Danio rerio* (zebrafish). Mammalian species were chosen based on the representatives available on ‘The Human Olfactory Data Explorer’ (HORDE) database (Olender, *et al.* 2013, Olender *et al.* 2020). Coding sequences of annotated sensory receptors were obtained from the NCBI RefSeq database (O’Leary *et al.* 2015).

For each species, Sensommatic predictions were mapped and aligned to their corresponding NCBI annotations with BLASTN. Three metrics were used to benchmark performance: recovery, prediction accuracy, and functional classification. Recovery was calculated as the percentage of NCBI receptors recovered, prediction accuracy as the percentage of predictions sharing 100% identity with their NCBI counterparts and functional classification as the proportion of recovered genes sharing functional status with their NCBI counterparts. Non-identical hits were further assessed to quantify differences and considered ‘improved’ if additional transmembrane domains or start/stop codons were incorporated. The mean score across these three metrics was used to quantify the *‘combined final score*’ (CFS) for each species.

Pipeline specificity was evaluated by quantifying the percentage of predictions representing sensory-specific GPCRs. DeepTMHMM (Hallgren *et al.* 2022) and the NCBI Conserved Domain Database search tool (Marchler-Bauer and Bryant 2004, Marchler-Bauer *et al.* 2010) were used to detect transmembrane domains, with TBLASTX (Altschul *et al.* 1990) searches against Uniprot (Bateman *et al.* 2022) and NCBI databases (Sayers *et al.* 2021) identifying putative non-sensory GPCRs. For visualization purposes, protein structure predictions were performed on representative receptors from each subfamily (Supplementary Figs S8 and S9) using ColabFold (Jumper *et al.* 2021, Mirdita *et al.* 2022).

Performance was assessed using three sets of tests (Tests 1–3). Test 1 evaluated performance when no adjustments are made to the reference files. For Test 2, species belonging to the same family as the query were removed from the reference file to assess how well Sensommatic recovers receptors in novel species. In Test 3, NCBI predictions for each species were used as reference files to test pipeline performance in an ideal self-annotation scenario. The mean CFS value across all 14 species was determined for each of these three tests and used to assess global performance in each testing scenario.

To assess performance relative to predictions from the HORDE database, we compared our OR predictions for human and dog to that of Olender *et al.* (2020). To account for the differences in genome assemblies used by Olender *et al.* (2020), two tests were performed for each species: running Sensommatic on the canFam3 (dog) and hg38 (human) assemblies used by Olender *et al.* (2020), and on the most recent version available for each species.

### 2.9 Evaluating pipeline run-times

To evaluate pipeline run-times, Sensommatic was run on the 14 test species and an additional 27 vertebrate species (Supplementary File S1). Genome assemblies were chosen based on varying assembly levels, with representatives from each class of vertebrates. Each test was executed with default parameters, using 20 threads with BLAST on a high-performance computing system equipped with dual Intel Xeon Gold 6152 processors (2.1 GHz, 22 cores each) and 384 GB of available memory.

## 3 Results

Sensommatic completed 39 of 42 tested assemblies within 8 h, with three reptile genomes taking up to 25 h to complete (Supplementary File S1). No significant correlation was found between run-time and the number of contigs in each query assembly (*R*^2^ = 0.0307, *P*-value = 0.1374), while a slight positive correlation was observed between run-time and the number of output receptors (*R*^2^ = 0.4001, *P*-value = 6.983e-06) (Supplementary Figs S3 and S4).

The mean CFS values for each of the three tests were 88.83%, 81.8% and 89.17%, respectively (Supplementary File S2 and Supplementary Fig. S5). For all three tests, the mean recovery (97.24%, 85.97%, 98.05%) and classification (94.91%, 90.76%, 93.04%) scores were high (Supplementary Fig. S6). We report high recovery scores in each test for functional receptors (99.11%, 88.09%, 98.86%). Sensory pseudogenes can be difficult to identify due to lineage-specific deterioration of the open reading frame and their generally poor representation in public repositories. We therefore report slightly lower recovery scores for pseudogenes (89.29%, 77.74%, 93.36%).

Prediction accuracy scores were significantly lower for pseudogenes than for functional receptors. We attribute this to the accumulation of non-sense and frameshift mutations, making them notoriously difficult to predict (Supplementary File S2). We also note that prediction accuracy may be artificially low for some species, arising from low quality NCBI predictions. For example, while 67.62% of predicted receptors were identical to NCBI predictions for duck in Test 1, 70.59% of Sensommatic’s non-identical predictions were found to be improved (additional transmembrane domains or start/stop codons identified) (Supplementary File S3). On average, 23.31% of non-identical predictions were of lower quality, while 18.95% were considered ‘improved’ relative to their NCBI counterparts across the 14 test species. Pipeline specificity was consistently high overall, with an average score of 98.75% across all tests (Supplementary File S4).

A performance loss was observed in Test 2 for frog when species belonging to *Pipidae* were removed from the reference file, with the mean CFS dropping from 86.13% in Test 1 to 22.55% in Test 2. To assess performance on other amphibians, we repeated Tests 1 and 2 on *Bufo bufo* and *Microcaecilia unicolor* (Supplementary File S2 and Supplementary Fig. S7). While CFS values remained stable for *M. unicolor* (88.01% Test1, 84.33% Test 2), we observe a similar performance drop in *B. bufo* (84.44% Test 1, 45.93% Test 2). We attribute this performance loss in *Anura* to the low taxonomic diversity in the Amphibian reference file, arising from the low availability of annotated amphibian genomes (Supplementary File S9) (Kosch *et al.* 2023). We aim to update the amphibian reference file as more annotated genomes become available.

When comparing Sensommatic predictions for dog to that of Olender *et al.* (2020), we recover 99.49% (canFam3) and 98.35% (canFam6) of ORs (Supplementary File S8). Moreover, we report an additional 102 receptors (38 functional, 64 pseudogenes) in canFam3 and 81 new receptors (26 functional, 55 pseudogenes) in canFam6 previously undetected. Notably, all 81 novel receptors (canFam6) are located on chromosome-level scaffolds and are unlikely to have arisen due to assembly errors. We therefore conclude that Sensommatic performs well on ‘dog’ in comparison to previous analyses. Applying Sensommatic to the human genome (hg38v39, hg38v40), we recover 94.23% of predictions from Olender *et al.* (2020), with 100% of functional receptors detected (Supplementary File S8). We detect 304 (hg38v39) and 380 (hg38v40) putative receptors not reported by Olender *et al.* (2020), 17 of which are located on chromosome-level scaffolds. Removing hits on unplaced scaffolds from our total count results in a final total of 818 OR genes detected for human (hg38v39, hg38v40).

## 4 Conclusion

Mining species-specific chemosensory repertoires allows for unprecedented insights into the evolution of perception. We have developed Sensommatic to utilize methods in genome mining/annotation for rapid recovery of sensory gene repertoires across vertebrates, accounting for unique lineage-specific sensory repertoires missed by traditional annotation methods and gene databases. The efficiency and full automation of the Sensommatic pipeline allows for its scalable application, removing current barriers to sensory receptor research, and facilitating large-scale investigations into sensory evolution in the era of reference-quality genomes.

## Supplementary Material

btae040_Supplementary_Data

## Data Availability

We have additional data that can be accessed on Figshare at: https://figshare.com/s/16212c123f783f1c14d0.
