# Supplementary material for: Sensommatic: an efficient pipeline to mine and predict sensory receptor genes in the era of reference-quality genomes
Source: Bioinformatics. 2024 Jan 23;40(1):btae040. doi: 10.1093/bioinformatics/btae040 (PMC10832353; doi:10.1093/bioinformatics/btae040)
Supplement: btae040_Supplementary_Data [file btae040_supplementary_data.zip › Revised_Supplementary_Methods.docx]

**Supplementary methods**

**Reference file generation**

All available RefSeq genome annotation files were downloaded from NCBI for each class of vertebrates. Species from the Vertebrate Genomes Project were excluded where the genome assemblies have not yet been published. The ‘coding sequence from genomic’ and ‘pseudogene without a product’ annotation files were downloaded for each species and mined for sensory receptors based on their assigned gene names. To compress the information in each reference file, and to decrease run-times, CD-HIT (Li, Jaroszewski and Godzik, 2001) was applied to cluster single exon genes in each reference file at 80% (mammals, Birds) or 90% (Reptile, Amphibian and Fish). Representative genes for each cluster were retained in the final reference files. Each reference file was checked against the NCBI Conserved domain database (CDD) (Marchler-Bauer and Bryant, 2004; Marchler-Bauer et al., 2010) for the presence of seven transmembrane domains. Receptors lacking transmembrane domains, or containing spurious domains, were filtered out. Each reference file was then scanned with deepTMHMM for transmembrane domains (Hallgren et al., 2022). Receptors containing less than four or more than seven transmembrane domains were removed. Receptors with lengths exceeding 3000 nucleotides were removed.

**Generating Profile HMMs**

Sensory receptors belonging to the class A GPCR superfamily (VN1Rs, OPNs, ORs and TAARs) and class C GPCR superfamily (VN2Rs, TAS1Rs and TAS2Rs) were mined from the RefSeq annotations for each of the fourteen test species. Class A receptors and Class C receptors were aligned with Clustal Omega (Sievers et al., 2014). The alignments were visualised and trimmed at both ends of the seven transmembrane region, with Jalview (Waterhouse et al., 2009). The adjusted alignments were used to build profile hidden Markov models (HMMs) of the seven transmembrane domains for Class A and Class C receptors using HMMER. Upon testing, functional type II vomeronasal receptors were consistently filtered out with the Class A and Class C profile HMMs. Therefore, a third profile HMM was generated for these type II vomeronasal receptors to increase recovery.

**Test set generation**

RefSeq coding sequences were mined for each species based on their sensory receptor gene annotations. Each receptor was mapped back to the genome with blastn (Altschul et al. 1990). Hits with 100% identity were considered a direct map, and assigned locus coordinates accordingly. Only one hit per locus was retained to remove splice isoforms. In-frame stop codons and sequence length were initially used to assign each receptor with ‘functional’ or ‘pseudogene_short’ status. RefSeq ‘pseudogene’ labels were retained regardless of sequence length. DeepTMHMM (Hallgren et al., 2022) was then applied to each test set. Receptors containing all 7 transmembrane domains were re-annotated as functional, except in sequences carrying in-frame stop codons, or where NCBI annotated the gene as a pseudogene. Sequences which lacked any transmembrane domains were removed from the test sets. Summaries are provided for each test set ([Supplementary File S5](https://docs.google.com/spreadsheets/u/0/d/1DBP2xGm_j6_gL5Hb7FrP9HEz9ExErE804L0xZIWDZsA/edit)).

**Self-annotation tests**

For the self-annotation tests (Test 3), each NCBI test set was filtered as described for the reference files. Reference genes with greater than 8 transmembrane domains, less than 4 transmembrane domains or with lengths greater than 3000 nucleotides were removed. The filtered test sets were used as both the reference file, and the test recovery set in Test 3 for each species.

**Calculating test scores**

Sensommatic predictions from each test species were mapped to their corresponding NCBI predictions using blastn. Genomic coordinates were compared to ensure each pair maps to corresponding loci. The number of Sensommatic predictions which map to NCBI receptors was used to quantify pipeline ‘recovery’. Hits which did not map to corresponding NCBI genes were considered ‘unmapped’ predictions. Similarly, NCBI receptors which did not map to any Sensommatic predictions were considered ‘missed’ predictions ([Supplementary File S6](https://docs.google.com/spreadsheets/u/0/d/1f3M-yfN3zTdKBqQcUuNANm7EwGsbwnrPQAF2cSpXsWA/edit))

To assess prediction accuracy, the 5’ and 3’ coordinates were compared for each pair. Pairs which shared start and stop positions were considered identical predictions. Where predictions were non-identical, the percentage of hits which over-extended, or did not extend enough, in the 5’ or 3’ direction was calculated ([Supplementary File S2](https://docs.google.com/spreadsheets/u/0/d/1BKPc5SF50cgSt2qobWzYCJUqpFefDkWMeM4E3KPqxzk/edit)). Furthermore, in situations where the 5’ and 3’ positions did not reconcile, the NCBI predictions were checked for the presence of start and stop codons. The percentage of discordant pairs, where the NCBI prediction lacked start and stop codons was quantified.

To quantify the percentage of hits which were correctly classified as ‘functional’ or ‘pseudogene’, the annotation status of each mapped pair was compared. The final score for each test was generated by calculating the mean percentage of recovered hits, perfect predictions and correctly annotated receptors.

**Quantifying prediction improvements**

To quantify the percentage of NCBI predictions which were improved by Sensommatic, DeepTMHMM (Hallgren et al., 2022) was applied to quantify the number of transmembrane domains in each mapped pair ([Supplementary File S3](https://docs.google.com/spreadsheets/u/0/d/1Etkao0tKa5LND9h6JklG9tD19d5XOpFOqHzbV3w8QBk/edit)). Sensommatic predictions were considered ‘improved’ where additional transmembrane domains were incorporated, or in cases where start or stop codons were included where lacking in the NCBI prediction. The same approach was used to determine the percentage of Sensommatic predictions which were worse than their NCBI counterpart.

**Validating specificity of unmapped predictions**

To assess pipeline specificity, unmapped predictions were translated and checked with deepTMHMM (Hallgren et al., 2022) for the presence of transmembrane domains. Where zero domains were detected, the predictions were further checked for transmembrane domains with the NCBI CDD search tool (Marchler-Bauer and Bryant, 2004; Marchler-Bauer et al., 2010). Hits with zero domains after deepTMHMM and NCBI CDD searches were deemed to be off target hits (Supplementary File S4).

To distinguish between sensory and non-sensory GPCRs, BLASTX (Altschul et al. 1990) searches were performed for each Sensommatic prediction against a custom GPCR database (Supplementary File S4). To construct this database, 79,891 non-sensory GPCRs from mammals were downloaded from the Uniprot database (Bateman et al. 2022) using the following keyword search: “GPCR AND (taxonomy_id:40674) NOT OLFACTORY NOT TASTE NOT VOMERONASAL NOT OPSIN NOT TAAR”. Additionally, 125,815 sensory GPCRs from mammals were downloaded from Uniprot using the following keyword search: “GPCR AND (taxonomy_id:40674) AND TASTE OR GPCR AND (taxonomy_id:40674) AND VOMERONASAL OR GPCR AND (taxonomy_id:40674) AND OPSIN OR GPCR AND (taxonomy_id:40674) AND OLFACTORY OR GPCR AND (taxonomy_id:40674) AND TAAR”. GPCRs downloaded from this search were flagged as “Sensory” and combined with the non-sensory GPCRs to comprise a combined dataset consisting of 205,706 GPCRs. If the top BLASTX hit for each Sensommatic prediction was a sensory GPCR, the prediction was considered to be a true sensory receptor.

Predictions which mapped best to non-sensory GPCRs in the Uniprot database, were considered “non-sensory candidates”. These non-sensory GPCR candidates were then searched against all vertebrate protein annotations on the NCBI database with BLASTX. As before, the top annotated hit was used to classify each candidate as ‘sensory’ or ‘non-sensory’. Predictions which were considered “non-sensory” after both blast searches were considered “off target hits”. The percentage of true sensory genes after deepTMHMM and BLASTX searches was used to quantify pipeline specificity (Supplementary File S4).

**Predictions filtered out with HMMER**

Predictions that failed to pass the HMMER filter were checked with deepTMHMM for the presence of transmembrane domains ([Supplementary File S7](https://docs.google.com/spreadsheets/u/0/d/1Ok1PtMfSKbezKkgcclje113wj7oGo48-71fYDhtjSJY/edit)). Hits which contain zero transmembrane domains were deemed to be off target hits which were filtered out with HMMER. Conversely, hits which contained transmembrane domains were considered to be good predictions which were removed inappropriately. The proportion of functional and pseudogenes filtered out with HMMER was also quantified.

**Pseudogene length optimization**

Sensommatic predictions are assigned pseudogene or functional status based on in-frame stop codons and prediction length. To determine the optimum length threshold for pseudogene classification, lengths of 850, 860, 870 and 900 were tested. The length with the greatest number of correctly classified predictions, when compared to the NCBI test sets, was found to be 860. Hence the default pseudogene length threshold is set to 860.

**Protein structure validation**

To validate Sensommatic predictions, protein structures were modelled with ColabFold using default parameters and visualised with Mol* 3D Viewer (Jumper et al., 2021; Sehnal et al., 2021; Mirdita et al., 2022). Representative predictions from each subfamily of sensory receptors were chosen from a diverse range of species. Receptors modelled include vomeronasal receptor type I (VN1R1) from *Monodelphis domestica,* olfactory receptor (OR6C3) from *Anas platyrhynchos*, taste receptor type II (TAS2R40) from *Anolis carolinensis*, trace amine associated receptor (taar14g) from *Danio rerio,* short wave opsin (opn1sw) from *Mus musculus,* medium wave opsin (OPN1MW) from *Homo sapiens,* long wave opsin (OPN1LW) from *Bos taurus* rhodopsin (RHO) from *Ornithorhynchus anatinus,*  type II vomeronasal receptor type I (VN2R26) from *Xenopus laevis* and type I taste receptor (TAS1R1) from *Canis lupus familiaris*. Protein structure modelling was also applied to verify novel prediction OR4F21 from *Pongo abelii.*

**Olender 2020 vs Sensommatic pairwise comparisons for human and dog**

Olfactory receptor predictions for human and dog were compared to those predicted in Olender et al., 2020. Due to differences in assembly versions used by Olender et al., 2020 (Supplementary File S8), four pairwise comparisons were carried out: CanFam3 Sensommatic vs CanFam3 Olender et al., 2020, CanFam6 Sensommatic vs CanFam3 Olender et al., 2020, hg38 v39 Sensommatic vs hg38 v39 Olender et al., 2020 and hg38 v40 Sensommatic vs hg38 v39 Olender et al., 2020. To compare results between Sensommatic predictions for CanFam6 and Olender et al., 2020 predictions for CanFam3, the USCS LiftOver tool was used to convert receptor coordinates from CanFam3 to CanFam6 (Supplementary File S8). A total of 100 receptors were removed in the conversion process. An additional 30 receptors were found to map to identical regions as other predictions, likely reflecting collapsed regions in the updated CanFam6 assembly. These 130 receptors were removed. Two human olfactory receptors (OR7E162P and OR7E103P) contained no sequence data in the fasta from Olender et al., 2020. In addition, there were no coordinates provided for six olfactory receptors (OR4C48P, OR4C49P, OR9G9, OR4C45, OR8U8 and OR8U9). Hence, these eight predictions were removed from the total olfactory receptor counts for human from Olender et al., 2020. Scores were calculated for each pairwise test as described above (Supplementary File S8).

**Supplementary References:**

Alföldi J, Di Palma F, Grabherr M *et al.* The genome of the green anole lizard and a comparative analysis with birds and mammals. *Nature* 2011;**477**:587–91.

Altschul SF, Gish W, Miller W et al. Basic local alignment search tool. Journal of Molecular Biology 1990;**215**:403–10.

Andrade P, Lyra ML, Zina J *et al.* Draft genome and multi-tissue transcriptome assemblies of the Neotropical leaf-frog Phyllomedusa bahiana. Kern A (ed.). *G3 Genes|Genomes|Genetics* 2022;**12**, DOI: <https://doi.org/10.1093/g3journal/jkac270>.

Bateman A, Martin M-J, Orchard S et al. UniProt: the Universal Protein Knowledgebase in 2023. Nucleic Acids Research 2022;**51**, DOI: https://doi.org/10.1093/nar/gkac1052.

Brashear WA, Bredemeyer KR, Murphy WJ. Genomic architecture constrained placental mammal X Chromosome evolution. *Genome Research* 2021;**31**:1353–65.

Church DM, Goodstadt L, Hillier LW *et al.* Lineage-Specific Biology Revealed by a Finished Genome Assembly of the Mouse. Roberts RJ (ed.). *PLoS Biology* 2009;**7**:e1000112.

Çilingir FG, A’Bear L, Hansen D *et al.* Chromosome-level genome assembly for the Aldabra giant tortoise enables insights into the genetic health of a threatened population. *GigaScience* 2022;**11**, DOI: https://doi.org/10.1093/gigascience/giac090.

Degani G, Nevo Sarel M, Hajouj A *et al.* Whole-Genome Inter-Sex Variation in Russian Sturgeon (Acipenser gueldenstaedtii). *International Journal of Molecular Sciences* 2022;**23**:9469.

Hallgren J, Tsirigos KD, Pedersen MD *et al.* DeepTMHMM predicts alpha and beta transmembrane proteins using deep neural networks. *bioRxiv* 2022, DOI: https://doi.org/10.1101/2022.04.08.487609.

Howe K, Clark MD, Torroja CF *et al.* The zebrafish reference genome sequence and its relationship to the human genome. *Nature* 2013;**496**:498–503.

Howe K, Dwinell M, Shimoyama M *et al.* The genome sequence of the Norway rat, Rattus norvegicus Berkenhout 1769 [version 1; peer review: 2 approved]. *Wellcome Open Research* 2021;**6**.

International Human Genome Sequencing Consortium. Initial sequencing and analysis of the human genome. Nature 2001;**409**:860–921.

Jones FC, Grabherr MG, Chan YF *et al.* The genomic basis of adaptive evolution in threespine sticklebacks. *Nature* 2012;**484**:55–61.

Jumper J, Evans R, Pritzel A *et al.* Highly accurate protein structure prediction with AlphaFold. *Nature* 2021;**596**:583–9.

Kalbfleisch TS, Rice ES, DePriest MS *et al.* EquCab3, an Updated Reference Genome for the Domestic Horse. *BioRxiv* 2018, DOI: https://doi.org/10.1101/306928.

Kirkness EF, Bafna V, Halpern AL *et al.* The Dog Genome: Survey Sequencing and Comparative Analysis. *Science* 2003;**301**:1898–903.

Köhler G, Vargas J, Than NL *et al.* A taxonomic revision of the genus Phrynoglossus in Indochina with the description of a new species and comments on the classification within Occidozyginae (Amphibia, Anura, Dicroglossidae). *Vertebrate Zoology* 2021;**71**:1–26.

Li W, Jaroszewski L, Godzik A. Clustering of highly homologous sequences to reduce the size of large protein databases. *Bioinformatics (Oxford, England)* 2001;**17**:282–3.

Li Y, Ren Y, Zhang D *et al.* Chromosome-level assembly of the mustache toad genome using third-generation DNA sequencing and Hi-C analysis. *GigaScience* 2019;**8**:giz114.

Lindblad-Toh K, Wade CM, Mikkelsen TS *et al.* Genome sequence, comparative analysis and haplotype structure of the domestic dog. *Nature* 2005;**438**:803–19.

Magallanes-Alba ME, Barricalla A, Rego N *et al.* Rapid genome functional annotation pipeline anchored to the House sparrow (*Passer domesticus*, Linnaeus 1758) genome reannotation. *BioRxiv* 2023, DOI: https://doi.org/10.1101/2023.01.27.525869.

Marchler-Bauer A, Bryant SH. CD-Search: protein domain annotations on the fly. *Nucleic Acids Research* 2004;**32**:W327–31.

Marchler-Bauer A, Lu S, Anderson JB *et al.* CDD: a Conserved Domain Database for the functional annotation of proteins. *Nucleic Acids Research* 2010;**39**:D225–9.

Mikkelsen TS, Wakefield MJ, Aken B *et al.* Genome of the marsupial Monodelphis domestica reveals innovation in non-coding sequences. *Nature* 2007;**447**:167–77.

Mirdita M, Schütze K, Moriwaki Y *et al.* ColabFold: making protein folding accessible to all. *Nature Methods* 2022;**19**: 679–682.

Moura Gama J, Ludwig A, Gazolla CB *et al.* A genomic survey of LINE elements in Pipidae aquatic frogs shed light on Rex-elements evolution in these genomes. *Molecular Phylogenetics and Evolution* 2022;**168**:107393.

Mouse Genome Sequencing Consortium, Waterston RH, Lindblad-Toh K *et al.* Initial sequencing and comparative analysis of the mouse genome. *Nature* 2002;**420**:520–62.

Myers EA, Strickland JL, Rautsaw RM *et al.* De Novo Genome Assembly Highlights the Role of Lineage-Specific Gene Duplications in the Evolution of Venom in Fea’s Viper (*Azemiops feae*). Qian W (ed.). *Genome Biology and Evolution* 2022;**14**, DOI: https://doi.org/10.1093/gbe/evac082.

Nakamura Y, Mori K, Saitoh K *et al.* Evolutionary changes of multiple visual pigment genes in the complete genome of Pacific bluefin tuna. *Proceedings of the National Academy of Sciences* 2013;**110**:11061–6.

Olender T, Jones TEM, Bruford E et al. A unified nomenclature for vertebrate olfactory receptors. BMC Evolutionary Biology 2020;**20**, DOI: https://doi.org/10.1186/s12862-020-01607-6.

Ren Y, Zhang Q, Yan X *et al.* Genomic insights into the evolution of the critically endangered soft‐shelled turtle Rafetus swinhoei. *Molecular Ecology Resources* 2022;**22**, DOI: https://doi.org/10.1111/1755-0998.13596.

Seabury CM, Dowd SE, Seabury PM *et al.* A Multi-Platform Draft de novo Genome Assembly and Comparative Analysis for the Scarlet Macaw (Ara macao). Janke A (ed.). *PLoS ONE* 2013;**8**:e62415.

Sehnal D, Bittrich S, Deshpande M *et al.* Mol* Viewer: modern web app for 3D visualization and analysis of large biomolecular structures. *Nucleic Acids Research* 2021;**49**, DOI: https://doi.org/10.1093/nar/gkab314.

Session AM, Uno Y, Kwon T *et al.* Genome evolution in the allotetraploid frog Xenopus laevis. *Nature* 2016;**538**:336–43.

Sievers F, Wilm A, Dineen D *et al.* Fast, scalable generation of high-quality protein multiple sequence alignments using Clustal Omega. *Molecular Systems Biology* 2014;**7**:539–9.

Streicher JW, Wellcome Sanger Institute Tree of Life programme, Wellcome Sanger Institute Scientific Operations: DNA Pipelines collective *et al.* The genome sequence of the common toad, Bufo bufo (Linnaeus, 1758) [version 1; peer review: 2 approved]. *Wellcome Open Research* 2021;**6**:281.

The Chimpanzee Sequencing and Analysis Consortium. Initial sequence of the chimpanzee genome and comparison with the human genome. *Nature* 2005;**437**:69–87.

Vine C, Teeling EC, Smith M *et al.* The genome sequence of the common pipistrelle, Pipistrellus pipistrellus Schreber 1774 [version 1; peer review: 2 approved with reservations]. *Wellcome Open Research* 2021;**6**.

Wang B, Ekblom R, Bunikis I *et al.* Whole genome sequencing of the black grouse (Tetrao tetrix): reference guided assembly suggests faster-Z and MHC evolution. *BMC Genomics* 2014;**15**:180.

Waterhouse AM, Procter JB, Martin DMA *et al.* Jalview Version 2--a multiple sequence alignment editor and analysis workbench. *Bioinformatics* 2009;**25**:1189–91.

Weissensteiner MH, Pang AWC, Bunikis I *et al.* Combination of short-read, long-read, and optical mapping assemblies reveals large-scale tandem repeat arrays with population genetic implications. *Genome Research* 2017;**27**:697–708.

Wiley G, Miller MJ. A Highly Contiguous Genome for the Golden-Fronted Woodpecker (Melanerpes aurifrons) via Hybrid Oxford Nanopore and Short Read Assembly. *G3 (Bethesda, Md)* 2020;**10**:1829–36.

Xu X, Arnason U. The mitochondrial DNA molecule of sumatran orangutan and a molecular proposal for two (Bornean and Sumatran) species of orangutan. *Journal of Molecular Evolution* 1996;**43**:431–7.

Zhou Y, Shearwin-Whyatt L, Li J *et al.* Platypus and echidna genomes reveal mammalian biology and evolution. *Nature* 2021;**592**:756–62.
