# Supplementary material for: Sensommatic: an efficient pipeline to mine and predict sensory receptor genes in the era of reference-quality genomes
Source: Bioinformatics. 2024 Jan 23;40(1):btae040. doi: 10.1093/bioinformatics/btae040 (PMC10832353; doi:10.1093/bioinformatics/btae040)
Supplement: btae040_Supplementary_Data [file btae040_supplementary_data.zip › Revised_Supplementary_Figures.pdf]

Supplementary Figures:

Supplementary Figure S1.
Sensommatic extension search for start and stop codons.

Supplementary Figure S2.
Sensommatic internal search for start and stop codons.

Supplementary Figure S3.
Pipeline run-time and the number of predicted receptors.

Supplementary Figure S4.
Pipeline run-time and the number of contigs in each genome assembly.

Supplementary Figure S5.
Pipeline scores per species for Tests 1-3.

Supplementary Figure S6.
Accuracy, specificity, classification and recovery scores per species for Tests 1-3.

Supplementary Figure S7.
Recovery scores *for Microcaecilia unicolor, Bufo bufo* and *Xenopus laevis* for Tests 1 and 2.

Supplementary Figure S8.
Protein structure validation of chemosensory receptors predicted with Sensommatic.

Supplementary Figure S9.
Protein structure validation of Sensommatic predictions for a type II Vomeronasal receptor and a type I Taste receptor.

Supplementary Figure S10.
Structural validation of novel Sensommatic prediction OR4F21 from *Pongo abelii.*

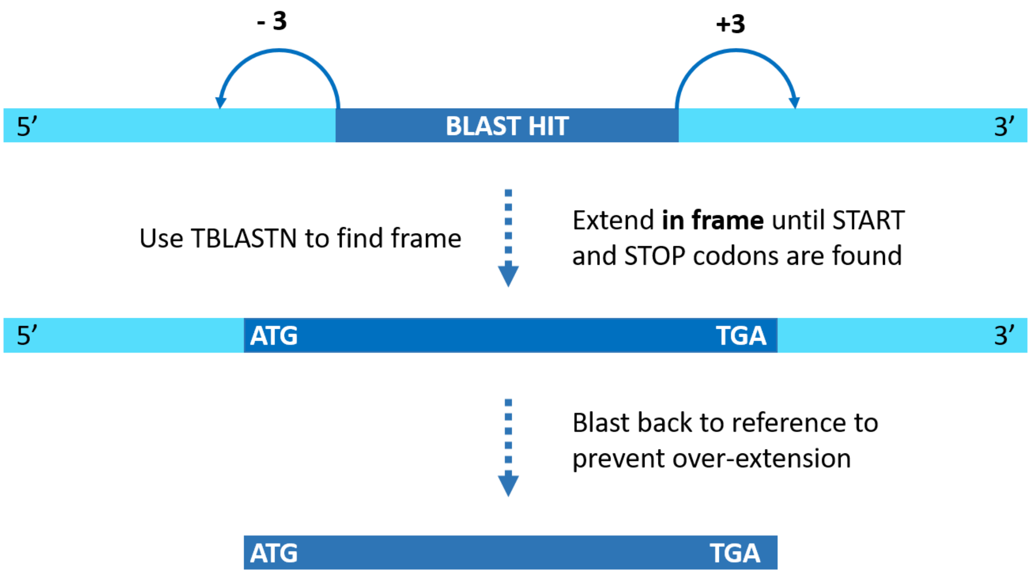

Supplementary Figure S1: **Sensommatic extension search for start and stop codons.** Single exon hits which fail to be predicted with AUGUSTUS undergo extension in frame to locate the start and end positions. A 'blastback' verification step is performed post extension to ensure over-extension has not occurred.

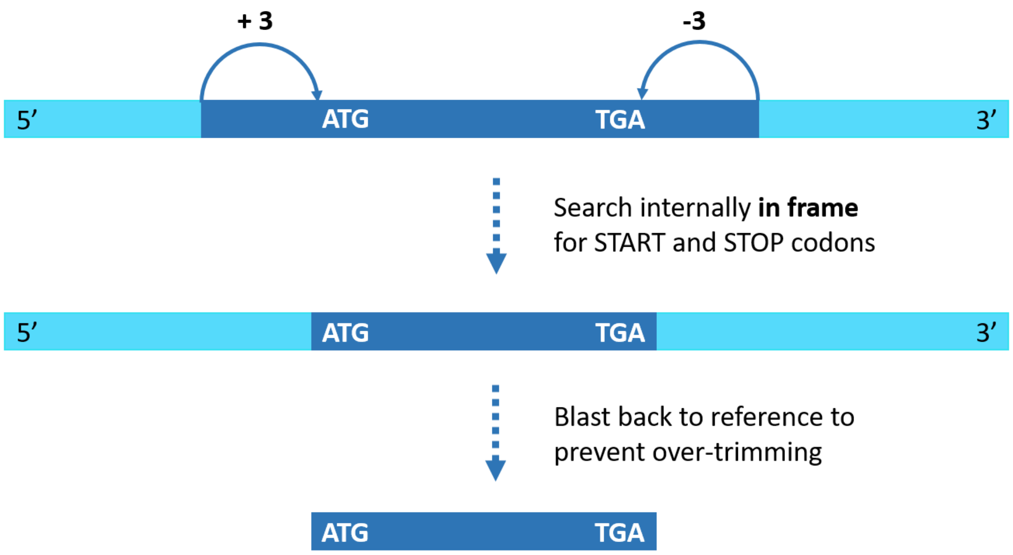

Supplementary Figure S2: Sensommatic internal search for start and stop codons. Single exon hits which fail to be predicted with AUGUSTUS, and which fail to locate the start and stop codons post extension, are cropped internally in frame to locate the start and end positions. A 'blastback' verification step is performed post the internal search to ensure over-contraction has not occurred.

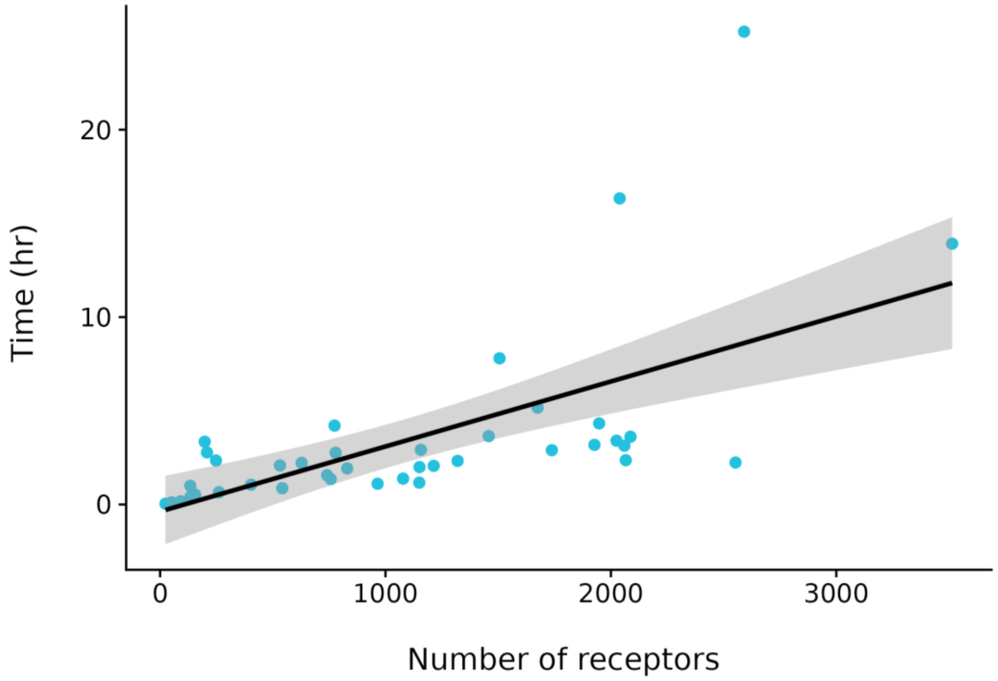

Supplementary Figure S3. Pipeline run-time and the number of predicted receptors. A slight positive correlation is observed (R^2 = 0.4001, p value = 6.983e-06).

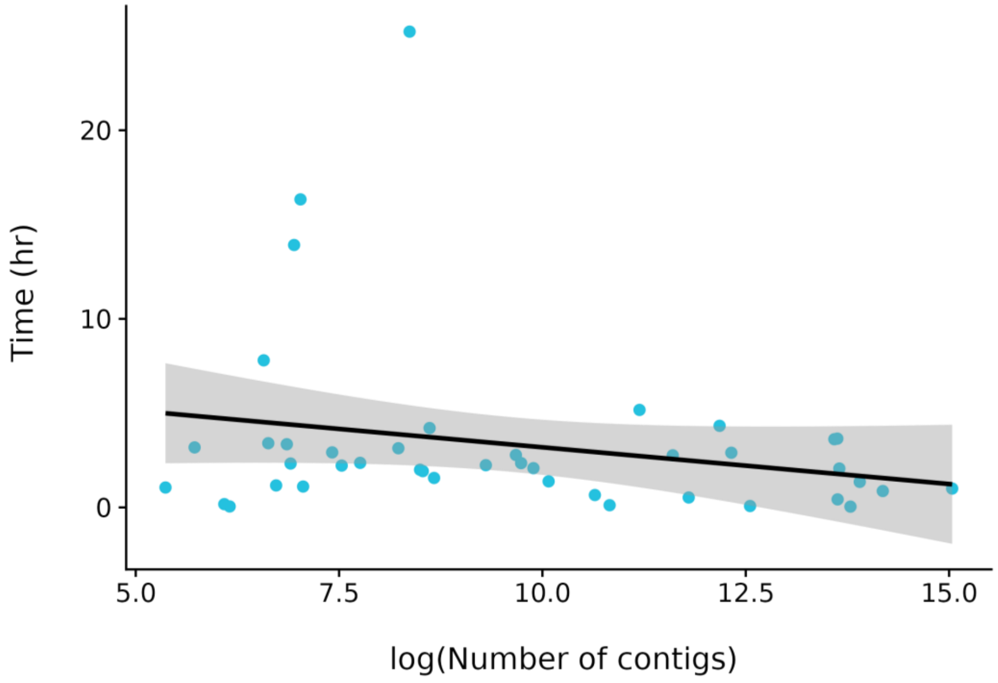

Supplementary Figure S4. Pipeline run-time and the number of contigs in each genome assembly. No significant correlation is observed (R^2 = 0.0307, p-value = 0.1374).

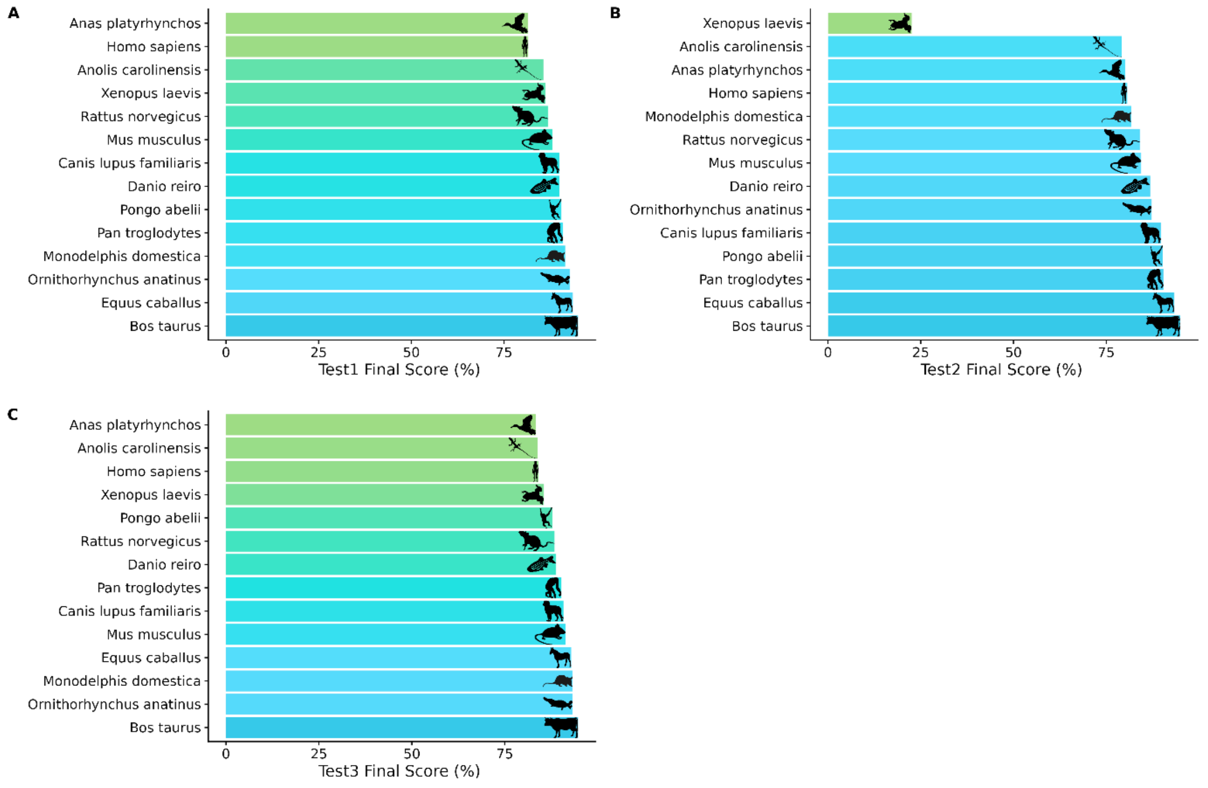

Supplementary Figure S5: Combined final scores per species for Tests 1-3. Prediction accuracy, recovery and functional classification were used to assess pipeline performance. The mean score across these three metrics was used to quantify the 'combined final score' per species as illustrated in this figure. In Test 1 (A), the default reference file was used. The query species was removed from the reference file in Test 2 (B). NCBI predictions for each query species was used as the reference file in Test 3 (C).

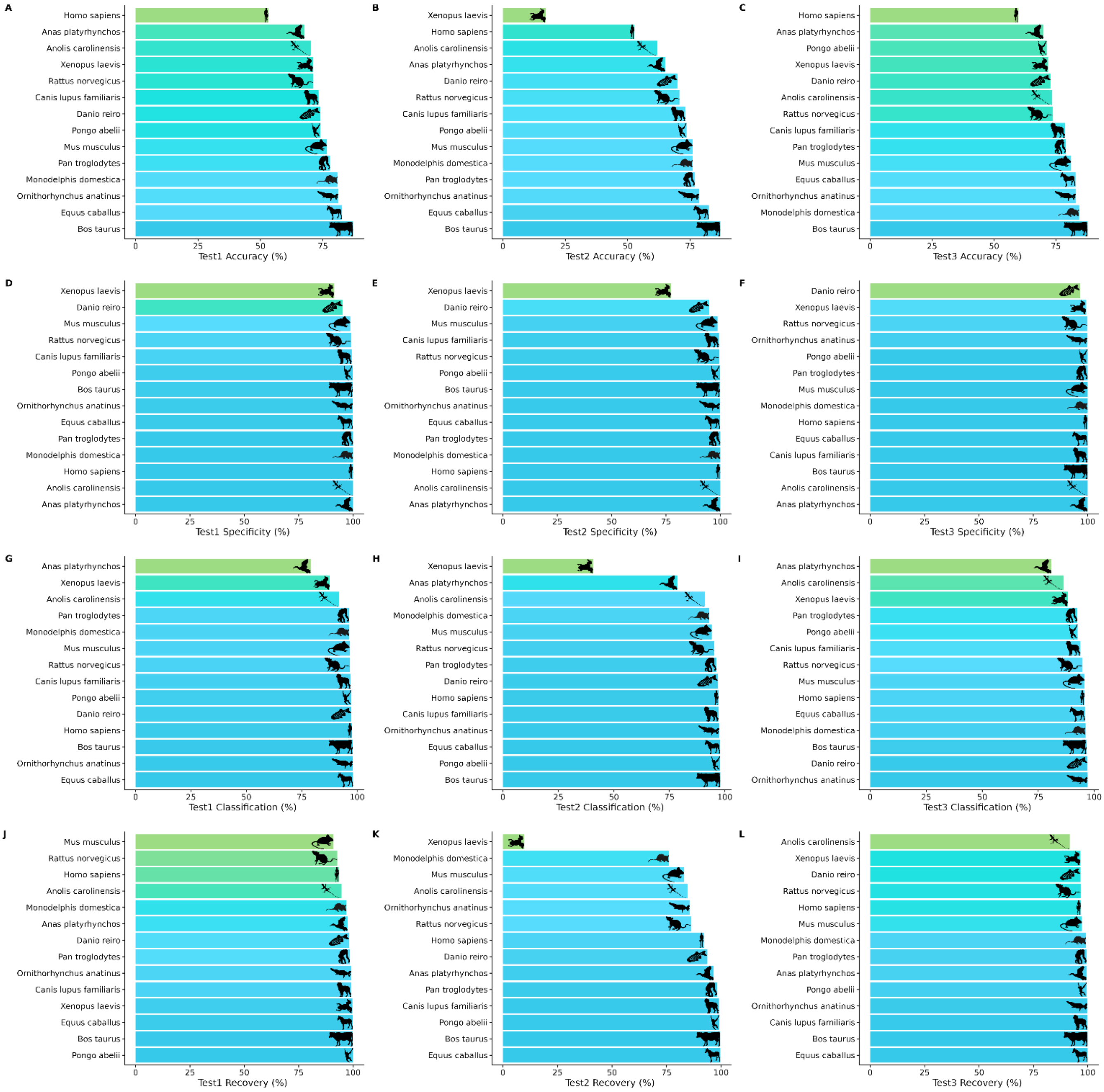

Supplementary Figure S6: Accuracy, specificity, classification and recovery scores per species for Tests 1-3. Percentage accuracy was quantified for Tests 1, 2 and 3 (A – C respectively) by finding the percentage of Sensommatic predictions which were identical to their NCBI counterparts. Specificity (D - F) corresponds to the percentage of hits which are verified sensory receptors. Percentage classification (G - I) corresponds to the percentage of genes correctly annotated with 'pseudogene' or 'functional' status. The number of NCBI genes recovered with Sensommatic was used to calculate percentage recovery (J - L).

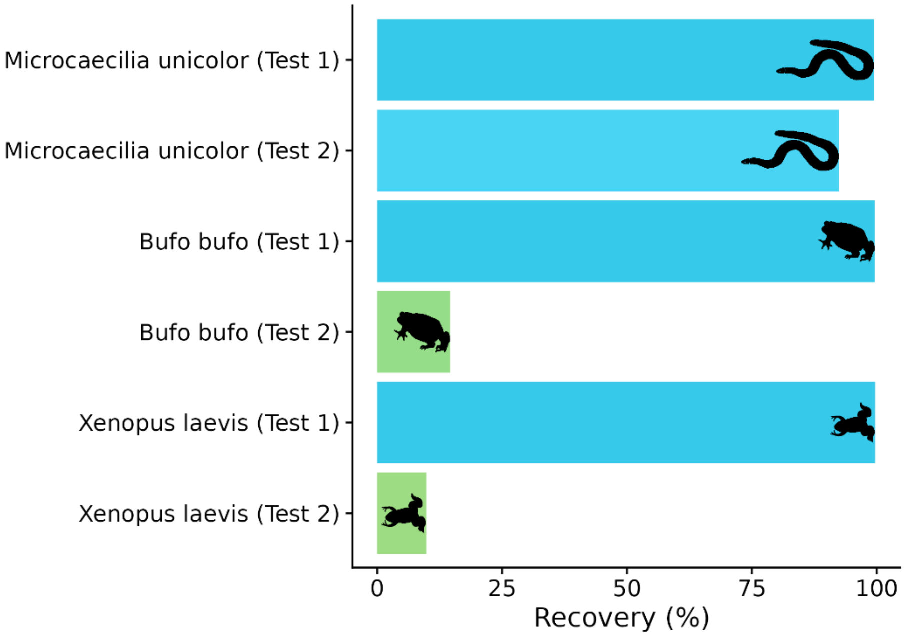

Supplementary Figure S7: Recovery scores for *Microcaecilia unicolor, Bufo bufo and Xenopus laevis* for Tests 1 and 2. A dramatic drop in recovery of receptors is observed for *Bufo bufo* and *Xenopus* laevis when the species from the representative family are removed from the reference file. Only a slight decrease in recovery is observed for *Microcaecilia unicolor* between Tests 1 and 2.

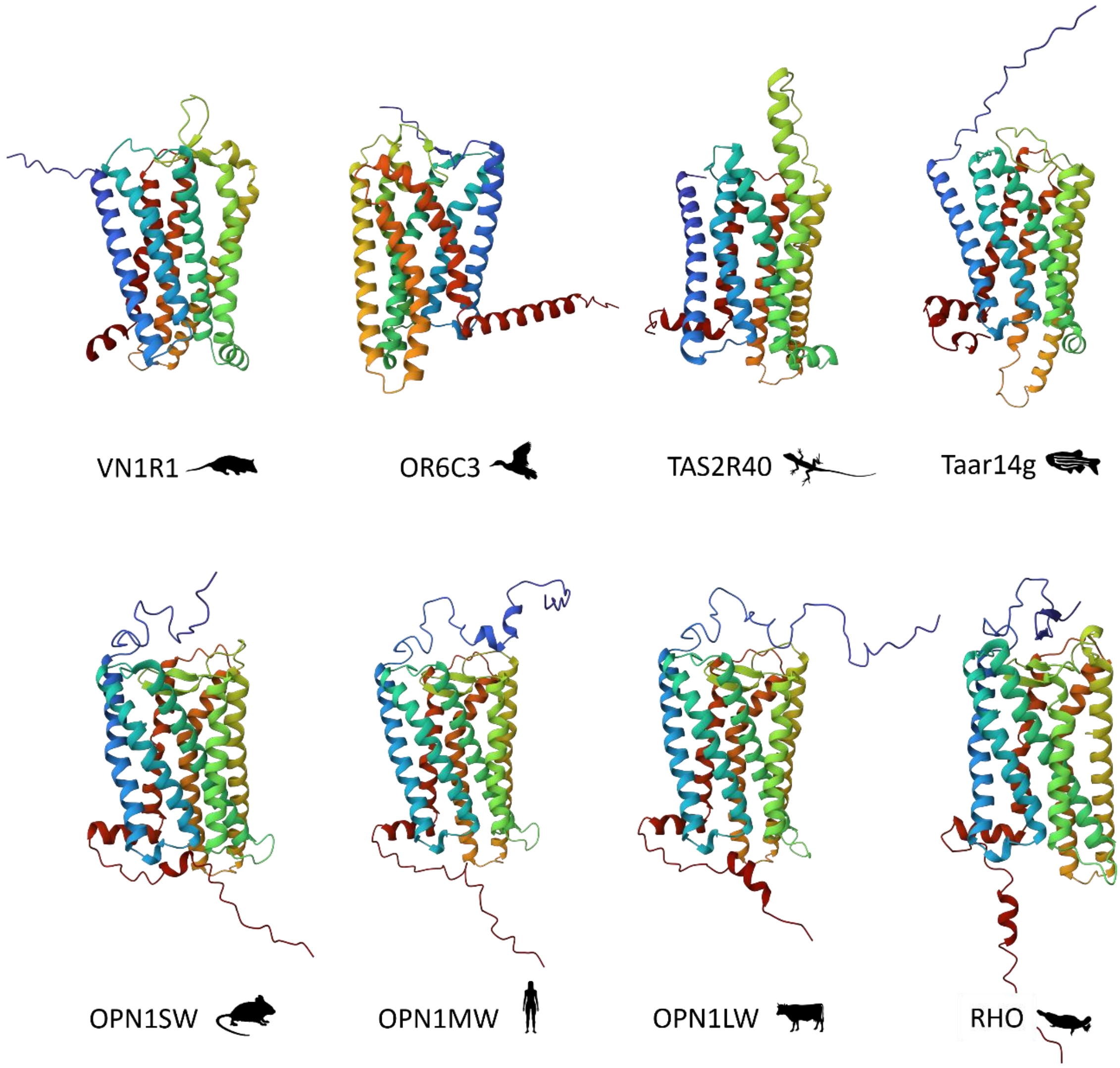

Supplementary Figure S8. Protein structure validation of chemosensory receptors predicted with Sensommatic. Protein structure predictions were generated with ColabFold and visualised with Mol* 3D Viewer (Jumper et al., 2021; Sehnal et al., 2021; Mirdita et al., 2022). Predictions are coloured from blue to red along the direction of the N to C terminus. The seven transmembrane domains can be seen in each prediction. Top row: vomeronasal receptor type I (VN1R1) from *Monodelphis domestica,* olfactory receptor (OR6C3) from *Anas platyrhynchos*, taste receptor type II (TAS2R40) from *Anolis carolinensis* and trace amine associated receptor (taar14g) from *Danio rerio.* Bottom row: short wave opsin (opn1sw) from *Mus musculus*, medium wave opsin (OPN1MW) from *Homo sapiens*, long wave opsin (OPN1LW) from *Bos taurus* and rhodopsin (RHO) from *Ornithorhynchus anatinus.*

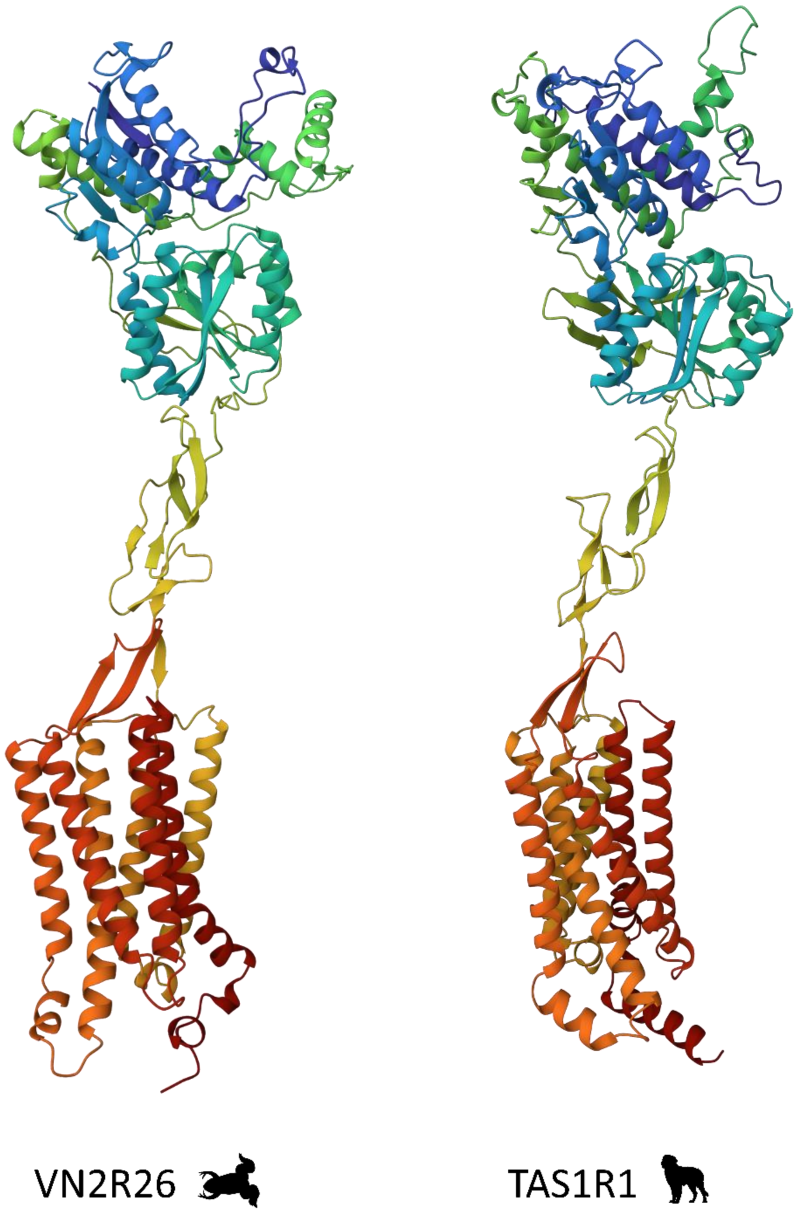

Supplementary Figure S9. Protein structure validation of Sensommatic predictions for a type II Vomeronasal receptor (left) and a type I Taste receptor (right). Protein structure predictions were generated with ColabFold and visualised with Mol* 3D Viewer (Jumper et al., 2021; Sehnal et al., 2021; Mirdita et al., 2022). Predictions are coloured from blue to red along the direction of the N to C terminus. Protein structures illustrated are for Sensommatic predictions of type II vomeronasal receptor (VN2R26) from *Xenopus laevis* and type I taste receptor (TAS1R1) from *Canis lupus familiaris*.

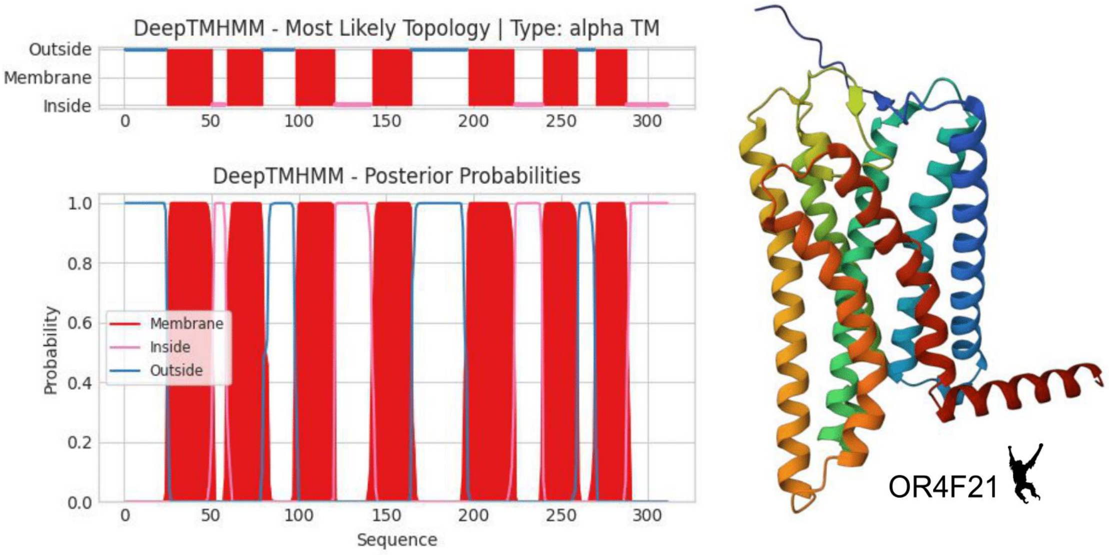

Supplementary Figure S10. Structural validation of novel Sensommatic prediction OR4F21 from *Pongo abelii*. The seven transmembrane domain structure is illustrated in red in the TMHMM plot (left), generated with deepTMHMM (Hallgren et al., 2022). Transmembrane domains are also visible in the protein structure (right), predicted with Colabfold and visualised with Mol* 3D Viewer (Jumper et al., 2021; Sehnal et al., 2021; Mirdita et al., 2022). The protein structure is coloured from blue to red in the direction of the N to C terminus. The genomic location of this prediction is 82379727 - 82380662 on chromosome 15 (NC_036918.1).
